# The Role of Serotype-Specific Immunological Memory in Pneumococcal Vaccination: Current Knowledge and Future Prospects

**DOI:** 10.3390/vaccines7010013

**Published:** 2019-01-29

**Authors:** Ioanna Papadatou, Irene Tzovara, Paul V. Licciardi

**Affiliations:** 1 Immunobiology and Vaccinology Research Laboratory, First Department of Paediatrics, Aghia Sofia Children’s Hospital, National and Kapodistrian University of Athens, 111527 Athens, Greece; iopapadatou@med.uoa.gr; 2 Murdoch Children’s Research Institute, Melbourne, VIC 3052, Australia; 3 Department of Paediatrics, University of Melbourne, Parkville, Melbourne, VIC 3010, Australia; paul.licciardi@mcri.edu.au

**Keywords:** pneumococcal vaccine, immunological memory, memory B cells, immune response, vaccination, *Streptococcus pneumoniae*, pneumococcal conjugate vaccine, pneumococcal polysaccharide vaccine

## Abstract

*Streptococcus pneumoniae* (*S. pneumoniae*, pneumococcus) is a major cause of morbidity and mortality worldwide. Achieving long-term immunity against *S. pneumoniae* through immunization is an important public health priority. Long-term protection after immunization is thought to rely both on protective serum antibody levels and immunological memory in the form of antigen-specific memory B cells (MBCs). Although the ability to achieve protective antibody levels shortly after pneumococcal vaccination has been well documented for the various infant immunization schedules currently in use worldwide, the examination of immunological memory in the form of antigen-specific MBCs has been much more limited. Such responses are critical for long-term protection against pneumococcal colonization and disease. This review summarizes the published literature on the MBC response to primary or booster immunization with either pneumococcal polysaccharide vaccine (PPV23) or pneumococcal conjugate vaccines (PCVs), aiming to elucidate the immunological mechanisms that determine the magnitude and longevity of vaccine protection against pneumococcus. There is evidence that PCVs induce the production of antigen-specific MBCs, whereas immunization with PPV23 does not result in the formation of MBCs. Increased understanding of the immunological factors that facilitate the induction, maintenance and recall of MBCs in response to pneumococcal vaccination could enable the use of MBC enumeration as novel correlates of protection against *S. pneumoniae*. Ongoing studies that examine MBC response to pneumococcal vaccination in high burden settings will be extremely important in our understanding of long-term protection induced by pneumococcal conjugate vaccines.

## 1. Introduction

*Streptococcus pneumoniae* (*S. pneumoniae*, pneumococcus) is a major cause of life-threatening infections, such as pneumonia, as well as invasive diseases including meningitis and sepsis, accounting for considerable morbidity and mortality worldwide [1].

Invasive pneumococcal disease (IPD) rates are higher in children less than 2 years of age, but remain significant throughout life, especially for individuals with immunocompromising and chronic diseases, as well as the elderly [1]. Acquisition of pneumococcal bacteria in the nasopharynx (colonization or carriage) is necessary for disease progression, but is often asymptomatic. However, in susceptible individuals, colonization can lead to dissemination of the organism to other sites in the body, leading to disease. Therefore, protection against colonization is a major strategy to protect against the development of pneumococcal disease.

Maintaining optimal levels of protection against *S. pneumoniae* throughout life is an important priority for public health policy-makers. Long-term protection after immunization is thought to rely both on protective serum antibody levels and immunological memory in the form of antigen-specific memory B cells (MBCs).

The first licensed pneumococcal vaccine was the 23-valent plain polysaccharide pneumococcal vaccine (PPV23) which has been used for the protection of immunocompromised individuals and the elderly against IPD and pneumonia for more than 2 decades [2]. The licensure of a plain polysaccharide vaccine (PPV) was based on trials of a 6-valent PPV and a 13-valent PPV [3,4], which showed strong vaccine efficacy against bacteremic pneumonia. In 1983, a 23-valent formulation containing a reduced 25 μg of each purified capsular polysaccharide replaced the earlier polysaccharide formulations, without, however, additional pre-licensure trials evaluating its efficacy against bacteremic pneumonia.

Today, two types of pneumococcal vaccines are available, each with different immunological characteristics and number of serotypes contained (Table 1): the PPV23, inducing a T-independent (TI) immune response with serotype-specific antibody formation but no immune memory, and a 10-valent (PCV10) and a 13-valent conjugated pneumococcal polysaccharide vaccine (PCV13), where pneumococcal polysaccharides are coupled with a carrier protein and therefore induce a T-dependent (TD) immune response [5]. While PPV23 induces only serotype-specific antibodies, PCV13 generates the formation of both serotype-specific antibodies and memory B cells, which are associated with longer duration of vaccine-induced immune responses. The first conjugate pneumococcal vaccine, 7-valent PCV (PCV7), was launched 18 years ago in the US and was rapidly incorporated in National Immunization Programs of many countries worldwide, but had to be later replaced by higher valency PCVs due to the increased incidence of IPD caused by non PCV7 serotypes (serotype replacement).

Infant PCV immunization programs were initially introduced as a 3 + 1 schedule in the US, based on two large studies that demonstrated high efficacy for this schedule [6,7]. Today, the World Health Organization (WHO) recommends the use of a 3-dose schedule of PCVs for the protection of children <2 years of age, and endorses PCV introduction in the routine immunization schedules of all countries, either as 3 primary doses without a booster (3 + 0) or as 2 primary doses with a booster dose (2 + 1) schedule [8]. The timing of these schedules, and particularly the use of a booster dose, has important implications in the generation of long-lived immunity that protects infants during a time of high susceptibility to this organism.

Although the high immunogenicity of these vaccination schedules in the form of antibody levels and opsonophagocytic activity measured shortly after vaccination has been repeatedly demonstrated in large vaccine trials [9,10,11,12], their ability to establish immunological memory in the form of antigen-specific MBCs has been much more poorly characterized.

However, recent changes in the global pneumococcal vaccination landscape may call for a closer look into the induction of memory B cells and their significance in the long-term protection of vaccinated populations.

As far as the developing world is concerned, a 3-dose primary PCV schedule without a booster has been recently introduced in most countries. This schedule has been found to be highly immunogenic, achieving high antibody titers during the first year of life [12]. Following implementation of the 3 + 0 infant schedule in African countries, substantial reductions were seen in infant morbidity and mortality. However, emerging data show that pneumococcal meningitis outbreaks continue to affect children older than 5 years of age and adults in the African meningitis belt [13,14], suggesting that the waning of immunity following such a schedule may be rapid and the achieved herd immunity is poor. Therefore, it is evident that high antibody levels post-vaccination alone may not translate into high vaccine efficacy in this setting and further characterization of the immune response might be necessary in order to achieve optimal protection through vaccination.

On the other hand, European countries with long-standing universal pneumococcal immunization schedules, such as the UK, aim to move towards reduced dosing schedules for infants [15]. In a recent study by Goldblatt et al. in the UK, it was shown that a 1 + 1 PCV13 schedule induces non-inferior antibody levels one month post-booster dose compared to the previously used 2 + 1 schedule, and it is estimated that the reduction of doses will not significantly affect the overall vaccine effectiveness due to the high coverage and the established herd immunity in this setting [16]. However, the recommendation of the country’s Joint Committee on Vaccination and Immunization to implement the 1 + 1 infant schedule into the National Immunization Program has raised concerns over the longevity of the conferred protection and the sustainability of herd immunity [17,18].

Therefore, it is evident that data on vaccine-induced immunological memory is essential in order to make educated shifts in immunization policies across different settings. The assessment of MBCs induced by the different vaccination schedules currently in use in the developing and the developed world could offer important information on the persistence of immunity and help inform policy makers on the most safe and efficient vaccination schedules to implement.

In this review, we aim to summarize the current knowledge on induction, maintenance, enrichment and recall of memory B cells in response to pneumococcal vaccination, in order to examine their role in the magnitude of the immune response and the longevity of vaccine protection.

## 2. Generation of Memory B Cells in Response to Vaccination: Current Knowledge

The production of high-affinity antibody-secreting cells is facilitated and regulated in the germinal centers (GCs), which are transient structures formed within the peripheral lymphoid organs in response to TD antigens. They typically consist of a central dark zone, where B cell blasts reside, and a peripheral light zone, which contains mainly T follicular helper (T_FH_) cells and follicular dendritic cells. In brief, upon vaccination with a TD-antigen, such as pneumococcal conjugate vaccine antigens, stimulated B cells enter GCs and undergo somatic hypermutation (SHM) of their B cell-receptor (BCR), producing clones with varying affinities for the immunizing antigen. Upon transit to the light zone, those clones with higher affinity are positively selected. The continuing recirculation of B cells between the two zones results in the production of high-affinity MBCs against the invading antigen [19,20,21,22].

**Figure 1:**
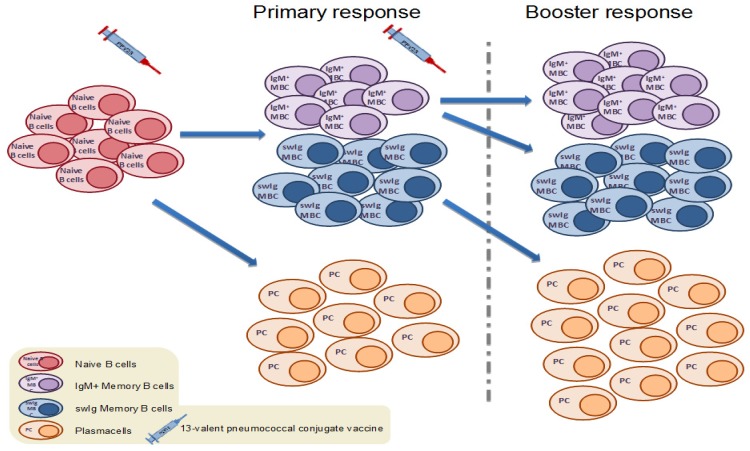
Upon primary vaccination with a pneumococcal conjugate vaccine (PCV), antigen-specific naïve B cells are activated following interaction with helper T cells. Some of the activated naive B cells differentiate into plasma cells, while others commit to memory and enter germinal center reactions with help from T follicular helper cells in order to generate antigen-specific IgM and switched memory B cells. Following antigen rechallenge, switched memory B cells enter rapid plasma cell differentiation, while IgM memory B cells (MBCs) enter secondary germinal center formation towards the formation of new IgM and switched memory B cells of higher affinity.

It has been recently proposed that different subsets of MBCs have different functions and immunological destinies. A number of studies have shown that human IgM MBCs seem to be the ‘guardians’ of immune memory, responsible for the replenishment of the B cell memory pool, entering GC reactions and generating new IgM and switched (swIg) MBCs upon secondary challenge with a TD vaccine antigen (Figure 1). At the same time, pre-existing swIg MBCs differentiate rapidly to antibody-secreting plasma cells [23,24,25].

This evidence suggests that levels of pre-vaccination IgM and swIg MBCs could predict the magnitude of memory B cell and humoral immune response to immunization with a TD vaccine, respectively. However, so far the relationship between MBCs and antibody concentrations at various intervals after immunization has been inconsistent between studies [26,27,28].

Further research towards understanding the distinct roles of the different components of the immunological memory pathway could provide the basis for more targeted and effective vaccination strategies. This would be of high importance in individuals with primary or secondary immunodeficiencies, conditions where different components of the immune response are compromised or absent.

Most of the information that we have on MBC induction, persistence and recall come from in vitro or animal model studies [23]. In the next sections, we review the methodology that has been developed in order to facilitate MBC studies in humans and we summarize the studies that have measured MBC responses to pneumococcal vaccines in various cohorts and settings (Table 2).

## 3. Enumeration of Human Antigen-Specific Memory B Cells in Peripheral Blood

Our ability to interrogate the MBC response following vaccination was facilitated by the development of a simple and convenient Enzyme-Linked Immunospot assay (ELISPOT) method by Crotty et al. in the early 2000s that allowed researchers to track human serotype-specific MBCs from peripheral blood mononuclear cells (PBMCs) [49]. Since then, this method has been used widely for the enumeration of MBCs against meningococcal and pneumococcal vaccine antigens [50].

Briefly, washed PBMCs previously cultured with polyclonal stimulators are seeded on specially designed ELISPOT plates coated with pneumococcal polysaccharides. Following co-incubation with anti-IgG or IgM antibodies and color, the reaction reveals spots in each well, which can be enumerated in an automated ELISPOT reader. Each spot represents a serotype-specific antibody-secreting cell derived from MBCs.

ELISPOT has numerous advantages; it is relatively easy to set up in the lab and perform with good reproducibility, and it is inexpensive and fast in comparison with other assays as it is possible to test for numerous antigens at the same time. Currently, this method is considered the gold standard for measuring antigen-specific B cell responses in a cost- and time-effective manner.

However, it should be kept in mind that the numbers of cells enumerated by ELISPOT do not correspond to actual numbers of circulating MBCs, since the culturing of PBMCs with polyclonal stimulators induces their proliferation and differentiation into antibody-secreting cells. Therefore, the output of the ELISPOT assay is a proxy for MBC numbers and therefore is not a direct measure of MBC frequency in the peripheral blood, but is nevertheless useful for the comparison of responses between individuals and time points in relation to vaccination.

Recently, an increasing number of studies report enumeration of MBCs induced by pneumococcal vaccination via Flow Cytometry. Methodology varies across these studies, mainly in terms of whether total or antigen-specific B cells are isolated (sorted) in the assay.

One technique for the detection of polysaccharide-specific B cells involves the biotinylation of pneumococcal polysaccharide (PS) antigens, which results in a biotin-PS conjugate [30,31]. This conjugate can be easily detected with an anti-biotin fluorochrome. With this technique, the biotin-PS conjugate is added to the cell suspension where it is bound by the PS-specific cells. The anti-biotin-fluorochrome then binds to and detects the cell–PS–biotin conjugate. Other variations of this technique have been developed where the PS is chemically pre-dyed with a fluorochrome and then added to the cell suspension to detect PS-specific cells [44,45,46,48,51]. Both techniques share a similar rationale and produce valid data.

Flow Cytometry may be more costly and laborious than ELISPOT, but MBC numbers reported correspond to actual frequencies in the peripheral blood of the vaccine recipients. Moreover, it enables not only the enumeration but also the phenotypic characterization of MBCs, offering a qualitative advantage compared to ELISPOT. Therefore, Flow Cytometry may also be a useful tool for elucidating the correlation between MBC and antibody response to vaccination and establishing MBCs as correlates of long-term vaccine protection.

## 4. Memory B Cells Response to Pneumococcal Vaccination

### 4.1. Memory B Cell Response to Immunization with the 23-Valent Plain Polysaccharide Pneumococcal Vaccine

**Figure 2:**
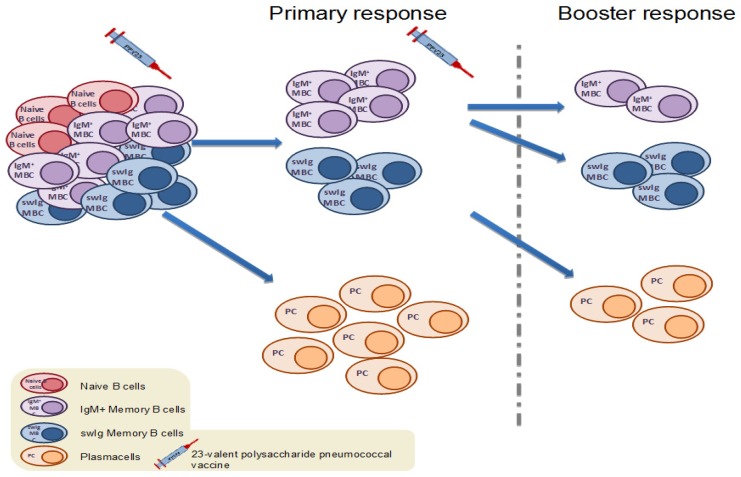
Upon primary vaccination with the 23-valent plain polysaccharide pneumococcal vaccine (PPV23), antigen-specific naïve B cells, as well as pre-existing antigen-specific MBCs formed by colonization or disease, are activated and differentiate into short-lived plasma cells (PCs) [52]. Booster immunization with PPV23 leads the remaining antigen-specific MBCs to terminally differentiate into PCs, thus resulting in further depletion of the antigen-specific MBC pool. This phenomenon is known as immune hyporesponsiveness [24].

**Figure 3:**
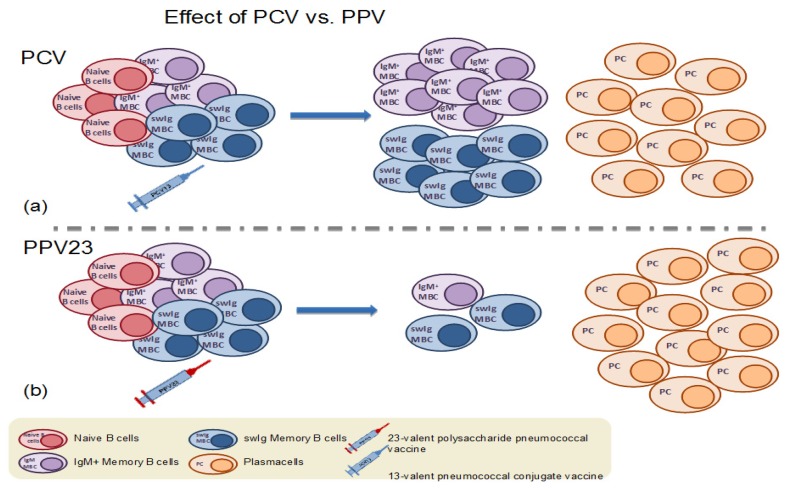
(**a**) Immunization with a dose of pneumococcal conjugate vaccine results in the formation of antigen-specific memory B cells and plasma cells. (**b**) In contrast, immunization with the 23-valent plain polysaccharide vaccine (PPV23) results in the depletion of the pre-existing antigen-specific memory B cell population.

The 23-valent plain polysaccharide pneumococcal vaccine has been known to induce a T-independent (TI), solely humoral immune response, with no ability to establish immunological memory. Theoretically, polysaccharide antigens, such as the pneumococcal antigens contained in PPV23 stimulate pre-existing MBCs towards terminal differentiation into antibody-secreting cells, thus resulting in the overall depletion of the memory cell pool and attenuated responses on re-exposure to the same antigen (Figure 2 and Figure 3). This PPV23-driven depletion of the MBC pool has been demonstrated in a small number of studies enumerating MBCs after PPV23 immunization (Table 2). In a recent study in HIV-infected adults, levels of IgM+ MBCs were significantly reduced following PPV23 vaccination [31]. In addition, in a study conducted in the UK, a dose of PPV23 resulted in a significant drop of serotype-specific MBCs in healthy adults and furthermore, MBC responses to subsequent immunization with PCV7, when given 6 months after PPV23, were attenuated [30]. In accordance, previous history of repeated immunizations with PPV23 in asplenic adults attenuated the MBC response to a dose of PCV13 in a dose- and time-dependent manner. Individuals with a history of more and recent PPV23s immunizations had consistently inferior MBCs and antibody counts post-PCV13 in comparison with those with fewer and earlier PPV23 immunizations in the past [37].

However, the PPV23-driven depletion of MBCs has not been a consistent finding across studies, as other research groups have found non-significant changes or even increases of MBCs after PPV23 immunization. In a recent study in Fijian children, there was no difference in MBC response to PCV13 in children who had or had not received PPV23 4–5 years ago [38]. Similarly, Indigenous Australians receiving repeated PPV23 vaccination 5 years apart demonstrated similar MBC numbers compared to Indigenous Australians who only received a single dose of PPV23 [43]. Most interestingly, a study in newly diagnosed HIV(+) adults showed significant increases in both IgM and swIg serotype-specific MBCs seven days after a dose of PPV23 for the two vaccine serotypes tested [45]. It has been postulated that pneumococcal polysaccharides might elicit the formation of non-classical MBCs generated outside of the germinal centers [52,53] through activation of marginal zone (MZ) and B1 cells which dictate the early response to TI antigens [54]. B1 cells are innate-like B cell subsets that have been better studied in mice and are thought to be responsible for the production of widely cross-reactive natural IgM antibodies [55]. Furthermore, mice studies imply that B1-b cells may be able to produce long-lived plasma cells in response to polysaccharides [52]. Studies in humans suggest that PPV23 may activate these cells leading to their differentiation into antibody-secreting cell (ASCs) and their subsequent depletion [30].

### 4.2. Memory B Cell Response to Primary Immunization with Pneumococcal Conjugate Vaccines in Infants and Children

Few data are available on MBCs induced by PCVs during a primary vaccination series in infants and children (Table 2). In a study by Clutterbuck et al. [29], it was shown that naive toddlers aged 12 months had no pre-existing swIg MBCs at baseline, suggesting that the amount of pneumococcal carriage experienced during the first year of life may not be sufficient to maintain detectable levels of swIg MBCs against pneumococcal serotypes in this age group. It was also shown that swIg MBCs increased significantly following a single dose of PCV7, although the MBC response in toddlers was lower than in adults. A second dose of PCV7 was necessary in order for toddlers to achieve MBC numbers similar to those mounted by adults after a single dose of the vaccine. Preliminary data from a randomized controlled trial of PCV schedules in Vietnam found that a 2-dose primary series schedule produced higher MBCs to selected serotypes compared with other schedules that incorporated a later booster dose [56].

### 4.3. Memory B Cell Response to Primary Immunization with Pneumococcal Conjugate Vaccines in Adults

A small number of studies (Table 2) have investigated the MBC primary response to PCVs in the adult general population as well as in high-risk groups such as immunocompromised individuals and the elderly. Accumulating data from these studies show that vaccine-naïve adults have pre-existing pneumococcal serotype-specific MBCs prior to immunization. This has been attributed to pneumococcal nasopharyngeal carriage and previous pneumococcal disease [29,36,37]. Significant increases 7 days and one month post-immunization with a single dose of PCV have been reported among healthy adults [29], adults with various immunocompromising conditions [31,37,51] and the elderly [29,30,37]. Interestingly, the levels of MBCs achieved post-immunization vary widely between different pneumococcal serotypes and thus may be affected by the immunogenicity of each antigen [29,37]. Age-driven immunosenescence [57] as well as immunocompromising conditions, such as HIV and asplenia, also diminish the MBC response in comparison to healthy young adults [58,59,60]. The severity of immunodeficiency, such as CD4 depletion in HIV infection, also affects the MBC response to PCV, as it has been shown that HIV+ patients with CD4 count <400/μL had lower MBC numbers than those with CD4 >400/μL one month post-PCV13 [31].

### 4.4. Memory B Cell Response to Booster Immunization with Pneumococcal Conjugate Vaccines in Children

By definition, memory cells are designed to be long-lived, remain in a steady state within the secondary lymphoid organs and, upon re-encounter with the same antigen, recirculate and differentiate rapidly to produce effector antibody-secreting cells, giving rise to a rapid and effective secondary immune response. Thus, the study of the MBC kinetics upon booster immunization is crucial for understanding the efficiency of immunological memory. A few studies have investigated the MBC response to booster vaccination with PCV following a primary PCV schedule; however, these studies vary in terms of number of the PCV doses included in the primary series and the interval between the completion of the primary schedule and the booster (Table 2). In a study in Fijian children previously immunized with a primary series of 0–3 doses of PCV7 in infancy and 0–1 doses of a PPV23 booster at 12 months of age, a dose of PCV13 at 5–7 years of age resulted in significant increases in serotype-specific MBCs for all 13 vaccine serotypes [38]. There was a trend towards lower MBCs pre-PCV13 in children who had received the PPV23 booster at 12 months compared to the PPV23-naïve; however, all children were able to mount similar numbers of MBCs one month post-PCV13 regardless of PPV23 vaccination history [38], suggesting that a dose of PCV at this age can overcome any previous PPV23-driven depletion of the memory B cell pool. Similarly, a study from the UK showed that a booster dose of PCV13 at 12 months of age induced significant increases of serotype-specific MBCs after a 2-dose primary PCV13 schedule at two and four months of age [40]. However, when a 10-valent PCV containing a different carrier protein was used as a booster instead of PCV13, MBCs did not increase one month post-booster, suggesting that the success of a booster response to PCVs may be dependent on homologous carrier protein priming [40]. In addition, in a recent study by the same group, a PCV13 booster resulted in significant increases of serotype-specific MBCs in toddlers and pre-schoolers aged 3–5 years who had been primed by either a 2-dose PCV7 or PCV13 schedule in infancy [39], demonstrating that a dose of PCV can induce booster responses in children even when administered years after the primary infant immunization. In another study, comparison of a booster dose at 11 months of age with PCV10 or PCV13 showed higher MBC responses for PCV13 for serotypes 6B, 7F and 9V but not serotype 1 (only 4 serotypes were examined) compared with PCV10, although the implications for these findings in terms of colonization and/or disease over the long-term are unknown [41].

### 4.5. Memory B Cell Response to Booster Immunization with Pneumococcal Conjugate Vaccines in Adults

Similarly to the kinetics seen in children, a booster dose of PCV administered at various intervals after the primary immunization results in significant MBC increases in adults. In a study by Clutterbuck et al., a second dose of PCV7 given 6 months after a primary dose resulted in further increase of serotype-specific MBCs [30]. Similarly, a dose of PCV13 given 7 years after immunization with PCV7 in asplenic adults with beta-thalassemia major induced significant increases in serotype-specific MBCs, despite previous history of multiple PPV23 immunizations, which is known to induce immunological hyporesponsiveness [37].

In contrast, an earlier study by Baxendale et al. in the elderly, showed only transient increases of IgG and IgA MBCs at one week post-immunization with a second dose of PCV7 given 6 months after the primary dose, but MBC numbers returned to baseline levels at one month post-booster [36]. Interestingly, post-immunization MBC numbers did not differ between recipients of one or two doses of PCV7, a dose of PPV23, or PCV7/PPV23 in this study, suggesting that pre-existing naturally-acquired immunity and age-related immunosenescence may blur MBC kinetics in the elderly.

### 4.6. Differences in Memory B Cell Response to Pneumococcal Vaccination Depending on the Recipient’s Health Condition

Very few studies have looked into the MBC response to either PCV or PPV23 vaccine in high risk populations with immunocompromising conditions [31,37,42,44,45,51]. The altered memory response to vaccination depends on the specific underlying immunological defect. In a study in asplenic young adults, a single dose of PCV13 resulted in significant increases of switched antigen-specific MBCs, while IgM MBCs remained at baseline levels [37]. The kinetics of IgM MBCs in this study could be affected by the lack of splenic marginal zone which has been proposed as the predominant source of circulating IgM memory cells [61].

Moreover, in a recent study, the response to PPV23 was diminished in HIV+ individuals compared to HIV-controls [32], suggesting that the HIV-induced dysfunction of the B cell compartment affects their ability to respond to TI antigens. Highly Active Antiretroviral Therapy (HAART) seems to restore B cell perturbations only partially, and a reasonable time after suppression of viral load is probably required for this restoration to occur [62,63]. Moreover, reduced numbers of CD4 T cells as a result of HIV infection lead to impaired TD responses. Thus, further investigation of the kinetics of B and T cells after anti-retroviral therapy is crucial for evidence-based recommendations regarding an optimal schedule and the timing of pneumococcal vaccination for HIV-infected individuals.

Finally, in a study in children with Down Syndrome, all B cell compartments, and especially switched MBCs, were reduced compared to their healthy siblings prior to booster immunization with PCV13 [42]. Booster immunization resulted in a significant increase of the swIg MBC pool in children with Down Syndrome which reached the MBC numbers of their siblings. It has been postulated that the reduced numbers of baseline swIg MBCs are due to their TLR9-driven exhaustion through increased terminal differentiation [42], although the ability of these children to form germinal centers seems to remain intact [64,65]. Thus, vaccination strategies that could sustain increased numbers of total MBCs may be able to outbalance their continuous loss.

The elucidation of the mechanism involved in each specific immunological impairment, along with the investigation of the different responses to vaccination in each immunocompromising condition, will help shape better vaccination policies for high-risk populations.

## 5. Correlation between Serotype-Specific MBCs and Antibody Responses

The relationship between memory B cell numbers and antibody responses remains controversial. Studies in animals and *in vitro* have shown that upon rechallenge with the same antigen, pre-existing antigen-specific IgM+ MBCs re-enter germinal center reactions in order to generate new memory cells with increased affinity, while swIg memory cells differentiate rapidly into plasma cells that secrete antibodies [23,24,25] (Figure 1). Based on this model, levels of pre-existing IgM+ MBCs should correlate with the MBC response post-booster immunization, while pre-existing swIg MBCs should correlate with the antibody response to the booster. However, data from different clinical studies have been inconsistent in regards to the MBC–antibody relationship.

In a study in healthy toddlers aged 1–2 years, swIg MBCs one month post a PCV13 booster were correlated with antibodies at the same timepoint and were also predictive of later antibody responses for some of the serotypes tested [40]. However, no significant correlation was found between baseline MBCs and antibody levels post a PCV13 booster in a study in older children conducted by the same group [39].

The lack of evidence for a consistent MBC–antibody relationship in human clinical studies could be partially attributed to the methodology of MBC enumeration used. Most of the earlier studies measuring MBCs before and after pneumococcal vaccination have used ELISPOT, a method that enumerates antibody-secreting cells derived from MBCs following culture and *in vitro* stimulation, as discussed earlier.

Memory B cell enumeration by Flow Cytometry may be more accurate in demonstrating actual MBCs frequencies in the peripheral blood of the vaccine recipients and thus, help to elucidate the correlation between MBCs and antibody response. However, application of Flow Cytometry in large PCV clinical trials in low and middle-income countries may be difficult due to the high cost of the assay and necessary equipment. In a recent study in HIV-positive adults, switched MBCs measured by Flow Cytometry at baseline were strongly correlated with antibody response at one month post-vaccination with one dose of PCV13 for both serotypes tested [31]. In the same study, pre-existing IgM+ MBCs were correlated with the level of swIg MBCs achieved post-vaccination [31].

## 6. The Potential of MBCs as Correlates of Protection

The enumeration of MBCs post-PCV immunization in the peripheral blood of humans is currently performed only for research purposes. The standardization of laboratory assays and the determination of the predictive value of MBCs for the persistence of humoral immunity and the magnitude of the booster response are necessary in order to establish memory B cells as correlates of protection with application in clinical practice. Up until now, antibody concentrations achieved after vaccination have been largely used for this purpose. It is plausible that MBCs may better predict long-term protection against nasopharyngeal carriage of the pneumococcus compared with current serological measures. This will be particularly important as we move into an area of reduced dose schedules involving a single primary and later booster dose (i.e., 1 + 1) as it is currently unknown how well these schedules protect over the longer-term. The results of ongoing studies in Vietnam as well as the UK, following the introduction of a 1 + 1 schedule, will be critical.

Quantification of the ability of a vaccine to establish immunological memory will enable physicians and researchers to make more precise evaluations of the various pneumococcal vaccination strategies.

## 7. Conclusions

In conclusion, PCVs are powerful vaccines that have demonstrated high efficacy against IPD as well as induced strong herd effects. The ability to predict the long-term effectiveness of PCVs against pneumococcal carriage and disease was not determined. Current measures of PCV-immunity, such as IgG serum antibody levels and opsonophagocytic activity, are used to compare the immunogenicity of different vaccines and schedules but their ability to predict long-term protection is more limited. Novel markers of PCV immunity such as MBCs may prove to be critical for this, and current studies that are aimed at examining this response in high burden settings will be extremely important in our understanding of the correlates of long-term protection induced by PCVs.

## Figures and Tables

**Table 1:** Formulation differences between licensed pneumococcal vaccines currently in use.

	**PCV10**	**PCV13**	**PPV23**
**Valency**	10-valent	13-valent	23-valent
Serotypes included	1, 4, 5, 6B, 7F, 9V, 14, 18C, 19F and 23F	1, 3, 4, 5, 6A, 6B, 7F, 9V, 14, 18C, 19A, 19F and 23F	1, 2, 3, 4, 5, 6B, 7F, 8, 9N, 9V, 10A, 11A, 12F, 14, 15B, 17F, 18C, 19A, 19F, 20, 22F, 23F and 33F
**Type**	Conjugated PS	Conjugated PS	Plain PS
Carrier protein (s)	TT with serotype 18C, DT with 19F, PD with all other serotypes	CRM with each serotype	none
Polysaccharide amount	1 μg/serotype (*3 μg/serotype for 4, 18C, 19F)	2.2 μg/serotype (*4.4 μg/serotype for 6B)	25 μg/serotype
Administration route	IM	IM	IM
**Target population**	healthy children	healthy children and adults >50 years of age	at risk population >2 years of age and adults >65 years of age

PCV10: 10-valent pneumococcal conjugate vaccine; PCV13: 13-valent pneumococcal conjugate vaccine; PPV23: 23-valent plain polysaccharide pneumococcal vaccine; PS: polysaccharide; PD: protein D from non-typeable *Haemophilus influenzae*; TT: Tetanus Toxoid; DT: Diphtheria Toxoid; CRM: CRM197 a non-toxic mutant of the diphtheria toxin; PCV7: 7-valent pneumococcal conjugate vaccine; IM: intramuscular.

**Table 2:** Characteristics of studies describing memory B cells following primary and booster vaccination with pneumococcal vaccines.

Reference	Population	Sample Size	Vaccine(s) Schedule	Method of MBC Enumeration
**(a) Response to primary immunization with PCVs**				
Clutterbuck et al. 2008, Clin. and Vac. Immunology [29]	adults 20–50 years and children 12m	60	1–2 PCV7	cultured ELISPOT
Clutterbuck et al. 2012, JID [30]	adults 50–70 years	150	2PCV7 + PPV23 or PPV23 + 2PCV7 or PCV7-PPV23-PCV7	cultured ELISPOT and Flow Cytometry
Farmaki et al. 2018, JID [31]	HIV+ adults	40	PCV13 + PPV23	Flow Cytometry
Ohtola et al. 2016, Vaccine [32]	HIV+ vs. healthy controls, 50–65 years old	51	PCV13 + PPV23 or only PPV23	Flow Cytometry
Truck et al. 2013, Immunobiology [33]	healthy adults 5–70 years	84	PPV23 or PCV7	cultured ELISPOT
Clutterbuck et al. 2006, Immunology [34]	healthy adults	10	1–2 PCV7	cultured ELISPOT
Kamboj et al. 2003, JID [35]	healthy adults, 22–35 years	24	PPV23 or PCV7	cultured ELISPOT
Baxendale et al. 2010, Vaccine [36]	healthy adults 50–80 years	37	PPV23 or PCV7	cultured ELISPOT
**(b) Response to booster immunization with PCVs**				
Baxendale et al. 2010, Vaccine [36]	healthy adults 50–80 years	37	PPV23 or PCV7	cultured ELISPOT
Papadatou et al.2014, CID [37]	asplenic adults (β–thalassemia), 19–48 years old	39	PCV13	cultured ELISPOT
Clutterbuck et al. 2012, JID [30]	adults 50–70 years	150	2PCV7 + PPV23 or PPV23 + 2PCV7 or PCV7-PPV23-PCV7	cultured ELISPOT and Flow Cytometry
Farmaki et al. 2018, JID [31]	HIV+ adults	40	PCV13 + PPV23	Flow Cytometry
Licciardi et al. 2016, J. Allergy Clin.Immun. [38]	healthy Fijian children	185	PCV13	cultured ELISPOT
Truck et al. 2017, Vaccine [39]	healthy children 3,5 years	62	PCV13	cultured ELISPOT
Truck et al. 2016, Ped.Inf.Dis. J. [40]	healthy children 1,2 years old	135	PCV10 or 13	cultured ELISPOT
van Westen et al. 2015, CID [41]	infants 1 year	104	PCV10 or PCV13	cultured ELISPOT
Valentini et al. 2015, Vaccine [42]	children with Down Syndrome vs. controls, 3–12 years old	30	PCV13	cultured ELISPOT
**(c) Response to immunization with PPV23**				
Licciardi et al. 2017, Clin. & Transl. Immun. [43]	Indigenous vs. non-indigenous Australians	60	PPV23	cultured ELISPOT
Iyer et al. 2015, J AIDS Clin. Res. [44]	HIV+ adults on HAART vs. HIV- controls	65	PPV23	Flow Cytometry
Leggat et al.2015, J AIDS Clin. Res. [45]	HIV+ newly diagnosed vs. HIV- controls	65	PPV23	Flow Cytometry
Leggat et al. 2013, Vaccine [46]	healthy adults, 24–30 years	17	PPV23	Flow Cytometry
Leggat et al.2013, JID [47]	elderly adults 64–88 years	14	PPV23	Flow Cytometry
Khaskhely et. al. 2012, J Immunol. [48]	healthy adults 18–30 years	22	PPV23	Flow Cytometry
Truck et al. 2013, Immunobiology [33]	healthy adults 5–70 years	84	PPV23 or PCV7	cultured ELISPOT
Kamboj et al. 2003, JID [35]	healthy adults, 22–35 years	24	PPV23 or PCV7	cultured ELISPOT
Baxendale et al. 2010, Vaccine [36]	healthy adults 50–80 years	37	PPV23 or PCV7	cultured ELISPOT

PCV7: 7-valent pneumococcal conjugate vaccine; PCV13: 13-valent pneumococcal conjugate vaccine; PPV23: 23-valent plain polysaccharide pneumococcal vaccine; HAART: Highly Active Antiretroviral Therapy; ELISPOT: Enzyme-Linked Immunospot assay.
